# Characterization of Two Novel Cell Lines with Distinct Heterogeneity Derived from a Single Human Bile Duct Carcinoma

**DOI:** 10.1371/journal.pone.0054377

**Published:** 2013-01-30

**Authors:** Jinghan Wang, Linfang Li, Keqiang Zhang, Yong Yu, Bin Li, Jiang Li, Zi Yan, Zhenli Hu, Yun Yen, Mengchao Wu, Xiaoqing Jiang, Qijun Qian

**Affiliations:** 1 Laboratory of Viral and Gene Therapy, Eastern Hepatobiliary Surgical Hospital, The Second Military Medical University, Shanghai, China; 2 The First Department of Biliary Surgery, Eastern Hepatobiliary Surgical Hospital, The Second Military Medical University, Shanghai, China; 3 Department of Molecular Pharmacology, Beckman Research Institute of the City of Hope National Medical Center, Duarte, California, United States of America; 4 Department of Respiratory, Changhai hospital, The Second Military Medical University, Shanghai, China; Virginia Commonwealth University, United States of America

## Abstract

**Background:**

Intratumoral heterogeneity reflects subclonal diversity and accounts for a variety of clinically defined phenotypes including the development of drug resistance and recurrence. However, intratumoral heterogeneity of bile duct carcinoma (BDC) is rarely studied.

**Methods:**

Two highly heterogeneous cell lines named EH-CA1a and EH-CA1b were established from a primary tumor tissue of a pathologically proven BDC. Distinct heterogeneity and underlying mechanisms of two cell lines in karyotype, colony formation, tumorgenicity, and sensitivity to chemoradiotherapy were intensively studied.

**Results:**

Both cell lines showed typical morphology of cancer cells. EH-CA1a cells grew as free-floating aggregates, while EH-CA1b cells grew adherently as a monolayer. EH-CA1a cells had higher cloning efficiencies and were able to keep proliferating under hypoxic condition. Coincidentally, hypoxia-induced factor-1α (HIF1α) and vascular endothelial growth factor (VEGF) mRNA were significantly higher in EH-CA1a cells than in EH-CA1b cells. Both cell lines were tumorigenic in nude mouse, however, EH-CA1a cells showed more aggressive characteristics. Most importantly, the EH-CA1a cells showed much more resistance against radiation and chemotherapy with gemcitabine. Metastasis-related genes including matrix metalloproteinase 2 (MMP-2), MMP-9, epithelial-mesenchymal transition (EMT) markers such as Vimentin, Snail, and Twist, are more highly expressed in EH-CA1a cells than in EH-CA1b cells. Moreover, the percentage of cells expressing cancer stem cell-like marker, CD133, in EH-CA1a cells is much higher than that in EH-CA1b cells. Moreover, knockdown of CD133 in both EH-CA1a and EH-CA1b cells significantly reduced their invasive potential and increased their sensitivities to radiation and gemcitabine, suggesting the differential expression of CD133 protein may partially account for the difference in malignancy between these two cancer cells.

**Conclusion:**

Establishment of these two cell lines will not only shed light on intratumoral heterogeneities of BDC, but also potentially facilitate the development of novel therapeutic approaches of BDC.

## Introduction

Bile duct carcinoma (BDC), a devastating malignancy arising from the bile duct epithelial cells, is the second most common primary hepatobiliary malignant diseases [1]. It has an annual incidence rate of 2 in 100,000 in the US (6000 new cases per year), and higher occurrence in northeast Thailand (85 in 100,000), China (7.55 in 100,000) and Korea (4.7 in 100,000) [2]. Previous studies have shown that BDC is a highly malignant carcinoma with heterogeneity in many aspects among different cases [3]. Clinical studies reveal that many patients have distinct responses to the same anti-cancer drug, which indicates that only small portion of patients have a chance to receive effective drug treatment. Even so, most of them still develop recurrence. Accumulating evidence support that tumor heterogeneity commonly exists at both the intratumoral and intertumoral level. Intratumoral heterogeneity relates to not only tumor recurrence, metastasis, but also resistance to chemoradiotherapy [4]. Many recent studies have identified extensive heterogeneity between individual tumors [5], [6] using large-scale sequencing analyses of solid cancers. However, tumor tissues within the same patient can also exhibit significant diversity. Genetic intratumoral heterogeneity has been shown and can contribute to treatment failure and drug resistance [7], [8]. Most recently, Gerlinger et al. have proved that spatially-distinct regions of the same clear cell renal carcinoma harbors heterogeneous somatic mutations and chromosomal imbalances, providing the molecular evidence for intratumoral heterogeneity [9].

The intratumoral heterogeneity of BDC remains unknown, and quantification of the heterogeneity remains a difficult task especially in those tumors without definite pathogenesis. Although we have found significant heterogeneity in BDC individuals already, intratumoral heterogeneity within single primary BDC tumors has not been systematically characterized yet [10].

In the current study, we successfully established and characterized two distinct bile duct cancer cell lines from the same tumor foci. Interestingly, these two cell lines display significant heterogeneity in many aspects such as morphology, growth pattern, invasiveness, metastatic potential, and genetics. Furthermore, the two cell lines have different sensitivity to hypoxia resistance and chemo-radiotherapy. The epithelial-mesenchymal transition (EMT), cancer stem cell markers, and cancer metastasis associated proteins such as Snail, Twist, CD133, and matrix metalloproteinase 2 (MMP-2), MMP-9 were differentially expressed in these two cell s. CD133 has been considered as an important cell surface marker for the subpopulation of cancer stem cells in many solid tumors [11]. Recent studies have also indicated that high expression of CD133 protein can serve as a prognostic indicator for tumor recurrence, metastasis, and patient survival [12], [13]. Additionally, high expression of CD133 also contributes to multi-resistance to chemoradiotherapy for many human cancers [14], [15]. Studies have also shown that EMT could promote stem cells properties and further generate cells with the features of tumor initiating property 16. EMT program also significantly maintained tumor initiating cells property 17. In hepatocellular carcinoma cells, expression of CD133 was also proved to be positively correlated with MMP-2 and a disintegrin and metalloproteinase (ADAM) 9 expression [18]. Consistently, in the present study we found that CD133, MMP-2/9 and other EMT proteins were more highly expressed in EH-CA1a cells, which had more malignant potentials compared with EH-CA1b. Furthermore, knockdown of CD133 in the two cells significantly lessened cell invasiveness and increased their sensitivities to radiation and gemcitabine. Taken together, the establishment of these two cell lines will shed light on intratumoral heterogeneities of BDC, and potentially facilitate the development of novel therapeutic approaches to BDC therapy.

## Materials and Methods

### Patient and Ethics Statement

This experimental protocol was approved by the Institutional Review Board of the Eastern Hepatobiliary Hospital, second military medical university, Shanghai, China. Written informed consent was obtained from the patient. All experiments handling human cells and tissues were performed in line with the tenets of the Declaration of Helsinki. The protocol was approved by Medical Experimental Animal Care Commission of Second Military Medical University. All surgery was performed under sodium pentobarbital anesthesia, and all efforts were made to minimize suffering.

A 52-year-old Chinese male was admitted to the hospital for surgical therapy as a result of full-body jaundice. Magnetic resonance imaging examination revealed intrahepatic and common bile duct expansion and a significant local stenosis. Serum biomarkers for HBV and HCV were negative, and α-fetoprotein (AFP) carcinoembryonic antigen (CEA) and carbohydrate antigen 19-9 (CA19-9) in blood was 3.5 ng/ml; 3.9 ng/ml and 228.9 U/ml, respectively.

### Isolation and Establishment of BDC Cell Lines

Post-surgery, about 50 mg of tumor tissue was randomly cut off from the central tumor area and washed twice with saline. Then tumor tissues were minced into 1 mm^3^ pieces and enzymatically disaggregated after incubating with collagenase type IV (Sigma-Aldrich, USA) solution at 37°C in a humidified atmosphere containing 5% CO_2_. After 45 minutes of incubation, a portion of a single tumor cell was isolated, and then 3 ml of fetal bovine serum (FBS) (Gibco, USA) was added to stop digestion. After the digested tumor fragments and fluid were filtered with a 200 mesh sieve, the filtrate was centrifuged at 1000 rpm for 5 minutes. The supernatant was removed and the remaining cells were resuspended in Dulbecco’s Modified Eagle Medium (DMEM) (Gibco, USA) supplemented with heat inactivated 10% FBS. The cells were seeded into a 6-well culture plate at a concentration of about 2×10^5^ per well, and then cultivated at 37°C in a humidified atmosphere containing 5% CO2. After several days of culturing, we found two distinct types of cells in the culture plate: spherical free-floating and polygonal adherent. In order to separate the floating cells from the adhesive cells, the culture medium was changed once a week. The floating cells can be extracted very easily by removing the medium. After centrifugation of old medium, cells were resuspended in new culture medium and seeded into new culture plate. The procedure was repeated ten times until the two distinct subpopulations of cells were completely separated. Following subculture, the cells were sampled at intervals, and frozen in liquid nitrogen; the stored cells could be propagated in culture without noticeable changes in growth or morphology. After 85 passages of EH-CA1a and 59 passages of EH-CA1b, the cell lines were designated EHCA1a and EHCA1b.

### Measurement of Secreted CA19-9, CEA, and AFP from Cancer Cells

10^5^ tumor cells were cultured in DMEM with 10% FBS medium for 24 hours before the conditioned medium was collected and centrifuged at 1800 rpm for 10 minutes. The supernatant was then collected for detection of CA19-9, CEA, and AFP by using the ELISA kit (R&D, USA) [10].

### Cytogenetic Analysis

Chromosome analysis, including G-banding was performed on EH-CA1a cells at passages-39 and on EH-CA1b cells at passages-23 as described before [19]. Colcemid was added to cell cultures with a final concentration of 0.25 mg/ml for 10–15 hours. Cells were then trypsinized and centrifuged at 1200 rpm for 12 minutes. Hypotonic medium of 0.075 M potassium chloride was used for 20 minutes at 37°C, and then the fixative (methanol/glacial acetic acid, 3∶1) was changed three times before slides were made. Some of the slides were aged at 60°C for 8–10 hours for G-banding. The aged slides were trypsinized for 3.5 minutes, rinsed in water, and stained with Giemsa, then rinsed again, air dried, and examined under the microscope.

### LOH/MSI Assay

In order to confirm the same origin of the two cell lines, microsatellite instability (MSI) and loss of heterozygosity (LOH) were performed using the LOH/MSI assay, as previously reported with slight modification [20]. Chromosome 8p, 13q, 16q and 17p are the sites of frequent loss of heterozygosity (LOH) or allelic imbalance (AI) in many different types of human cancers, including liver cancer, breast cancer, prostate cancer, bladder cancer, as well as oral and laryngeal squamous carcinoma [21]. So here we selected five microsatellite markers including D8S264 (141 bp), D8S277 (121 bp), D13S268 (298 bp), D16S505 (239 bp), and D17S831 (230 bp), which locate on 8p23, 8p23, 13q14, 16q24 and 17p13, respectively. PCR reactions and gel electrophoresis were performed as described previously [20]. The MSI score was calculated using the electrophretic mobility shift assay (EMSA). A positive score for comparison between EH-CA1a and EH-CA1b DNA products indicate that a shift was seen in at least one of the five loci tested. The results were also confirmed by an additional repeat of the PCR and electrophertic procedure. LOH was scored only if the radiographic signal was clearly reduced in one of the cell’s DNA.

### Cell Proliferation and Cell Cycle Assays

The cell growth curve was determined by MTT (3-(4, 5-dimethylthiazolyl-2)-2, 5-diphenyltetrazolium bromide) assay. EH-CA1a (passage-38) and EH-CA1b (passage-36) cells were plated into 96-well plates at 4,000 cells per well and cultured in 15% FBS DMEM for various durations. Cell proliferation rate was measured by MTT assay according to the manufacturer’s protocol. The doubling times were determined from the growth curve. Cell proliferation under hypoxic environment (1% O_2_) was also measured by the method above.

Cell cycle analysis was performed after fluorescence labeling the cellular deoxyribonucleic acid (DNA) with propidium iodide (PI) (Invitrogen, Carlsbad, CA, USA). After two cold PBS rinses, EH-CA1a (passage-38) and EH-CA1b (passage-36) (1×10^5^) were harvested during the exponential growth phase, fixed with cold 70% alcohol, and then incubated for 4 hours at 4°C. After centrifugation at 1000 rpm for 3 minutes and cold PBS rinse, cells were resuspended in 1 ml propidium iodide stain (50 µMol/L propidium iodide, 100 µg/ml RNase) and incubated for 30 min at room temperature. The cells were then sent for cell cycle analysis by FACS (BD, US).

### Anchorage-independent Growth Assay in Soft Agar

Anchorage-independent growth was assessed by suspending 1×10^5^ EH-CA1a (passage-40) and EH-CA1b (passage-38) cells in 1 ml 0.3% (w/v) soft agar (Detroit, MI, USA) layered over 0.3% (w/v) base agar in 24-well plates; agar was supplemented with DMEM and 10% FBS. Colonies containing >50 cells were scored and pictured by the inverted microscope at 2 weeks post-seeding.

### Invasion Assay

To investigate the cells’ invasive abilities, we used transwell chamber with an 8 µm pore membrane filter (Corning NY, USA) coated with 50 mg of matrigel in a 24-well culture plate. EH-CA1a (passage-35) and EH-CA1b (passage-30) cells were resuspended at a final concentration of 1×10^4^ cells per ml in 0.1 ml DMEM with 1% FBS in the upper chamber. The lower chambers were loaded with 0.6 ml medium containing 10% FBS. After incubation for 4 hours in an atmosphere of 37°C with 5% CO_2_, cancer cells on the upper surface of the membrane were removed and stained with trypan blue. Cancer cells that invaded through the matrigel-coated filter on the lower membrane were manually counted under a microscope. Four randomly chosen fields were counted for each assay.

### Labeling the EH-CA1a and EH-CA1b Cells with Firefly Luciferase Reporter Gene

The lenti-luc2 vector system was purchased from Promega Corporation (Promega, WI, USA). High-titer lentivirus production and lentiviral transduction of cancer cells were performed as previously reported [22]. The lentivirus infected cancer cells were further selected at 1 µg/ml of puromycin to generate EH-CA1a-luc and EH-CA1b-luc cell lines stably expressing luciferase. The high expression of luciferase in both cell lines was confirmed by imaging of the cells with fluorescent microscopy (FluoView FV1000, Olympus).

### Mouse Xenograft Model of EH-CA1a-luc and EH-CA1b-luc Cells

The protocols for *in vivo* mouse xenograft models were approved by the Second Military Medical University’s Medical Experimental Animal Care. The surgeries were performed under sodium pentobarbital anesthesia, and all efforts were made to minimize suffering. 6-week-old male BALB/c nude mice were provided by the Shanghai SLAC Laboratory Animal Co. Ltd of Chinese Academy of Sciences, and the mice were maintained in a specific pathogen-free condition. EH-CA1a (passage-40) and EH-CA1b (passage-38) cells (1×10^6^ cells) mixed with 0.2 ml saline was injected subcutaneously into the animals’ backs. Three weeks later, all subcutaneous tumors were removed, weighted, and fixed in 10% formalin in preparation for paraffin sections before staining them with haematoxylin and eosin (H&E). P-values of *P<0.05* were considered to be statistically significant.

The metastatic tumor growth was measured by luciferase activity detection using the In Vivo Imaging System (IVIS). For the experimental metastasis assay, a total of 2.5×10^6^ EH-CA1a-luc (passage-30) and EH-CA1b-luc (passage-28) cells were injected into the abdominal cavity of the mice separately. Successful injections were confirmed by immediate IVIS detection. Two weeks later, the colonization of cancer cells in mice was monitored by the animal IVSI.

### Sensitivity to Chemoradiotherapy

The effects of anti-cancer drugs or radiation treatment on the survival of EH-CA1a (passage-45) and EH-CA1b (passage-38) cells were measured using 3-(4, 5-dimethylthiazole-2-yl)-2, 5 diphenyltetrazolium bromide (MTT) assays as previously described with slight modifications [23]. After seeding approximately 1×10^4^ cells into 96-well culture plates for 24 hours, various concentrations of gemcitabine (Eli Lilly), 5-Fu, and sorafenib (Nexavar) were added to the culture medium. 72 hours after drug treatment, the cells were then incubated with 20 µL of MTT (5 mg/ml) for 4 hours at 37°C, and 200 µL of DMSO was added to precipitate the crystals for 20 minutes at room temperature. The optical density was determined with a spectrophotometer (Bio-Rad, USA) using wavelength of 570 nm. The overall experiment was repeated three times. The cell survival rate (percent) is calculated using: survival rate = (1−OD_drug_/OD_control_) × 100% where OD_drug_ and OD_control_ are the OD values of the groups with and without anticancer drug treatment, respectively. For the clonogenic survival assay, cells were plated onto 25 cm^2^ tissue culture flasks (Corning, MA) at concentration of 15,000 cells per well, and then exposed to irradiation from a ^137^Cs source for time periods equivalent to 2.0, 4.0, 6.0, 8.0, and 10.0 Gy. After 14 days, cells were washed with PBS, fixed with 10% acetic acid and 20% ethanol solution, and stained with 4% crystal violet. Washed dishes were dried, and colonies containing >50 cells were scored. The assays were repeated at least three times.

### Total RNA Isolation and Quantitative Real Time-PCR

Total RNAs were extracted from EH-CA1a (passage40) and EH-CA1b (passage38) cells using the SV Total RNA isolation system (Promega, USA). The cells were also cultured in 1% O_2_ condition for 24 hours, followed by total RNA extraction of EH-CA1a (passage-40) and EH-CA1b (passage-38). First-strand complementary DNAs (cDNAs) were generated using the qScript™ cDNA SuperMix kit and protocol by Quanta BioSciences (MD, USA). Quantitative real-time Polymerase Chain Reaction (RT-PCR) analyses were performed using the SYBR Green Real-time master mix (ToYoBo, Japan) with the primers listed in Table 1. All experiments were independently performed three times. Results were presented as means ± SD, and were compared using the Student’s t-test and ANOVA. *P<0.05* was considered to be statistically significant.

**Table 1:** Primer Sequence.

Primer name	Primer sequence(5′-3′)
Snail	F:TTCTTCGCTACTGCTGCG
	R:GGGCAGGTATGGAGAGGAAGA
E-cadherin	F:TGATGCCCCCAATACCCCAG
	R :CTGTGGAGGTGGTGAGAGAG
Integrin α5	F:ACCAAGGCCCCAGCTCCATTAG
	R :GCCTAACACTGCAGGCTAAATG
Twist	R:GGGAGTCCGCAGTCTTACGA
	R:CTAGTGGGACGCGGAC
MMP-2	F:CACCATCGCCCATCATCA
	R:TGGATTCGAGAAAACGCAGC
HIF-1α	F:GAGGAAATGAGAGAAATGCTTACA
	R:GCTTCGCTGTGTGTTTTGTTCTT
VEGF	F: CGAAACCATGAACTTTCTGC
	R: CCTCAGTGGGCACACACTCC
GAPDH	F:TGGTATCGTGGAAGGACTCATGAC
	R:ATGCCAGTGAGCTTCCCGTTCAGC

### Western Blot

The mouse monoclonal antibodies against GAPDH, rabbit monoclonal antibody against human VEGF and Twist were obtained from Santa Cruz Biotechnology (Santa Cruz, CA). Rabbit monoclonal antibody against human HIF1α was purchased from BD Transduction Laboratories (BD Biosciences, CA). Total cell lysates (40 µg proteins) were separated using 10% SDS–PAGE gels and transferred onto polyvinylidene difluoride membranes (Bio-Rad Laboratories, CA, USA). After blockage with 5% milk, the membranes were incubated with primary antibody (1∶1000 dilutions) overnight at 4°C. The membrane was washed and then incubated with indicated secondary antibodies for 1 hour. Immunoreactive proteins were detected with enhanced chemiluminescence reagents (Pierce, Rockford, IL, USA) and photographed with Kodak X-Omat blue autoradiography film.

### Cancer Stem Cell Surface Marker Expression Analysis

Cells (10^6^) were trypsinized and suspended in 100 ml D-Hank’s buffer. APC or FITC-labeled anti-bodies targeting cell surface markers CD90, EpCAM, and CD133, were added to cell suspension and incubated on ice for 20 minutes. Cells were then washed with D-Hank’s buffer twice, re-suspended in 300 µl D-Hank’s buffer, and sent for FACS analysis (Becton Dickinson, Mountain View, CA, USA). Each experiment was repeated three times using cells from different passages.

### Small Interference RNA (siRNA) Transfection

Control siRNA and human CD133 specific siRNA were purchased from Life Technologies™ (Foster City, CA). CD133 knockdown was performed according to the Lipofectamine RNAiMAX (Invitrogen) manufacturer’s protocol. Briefly, 1×10^5^ cells were seeded in 6-well plates and cultured in DMEM medium supplemented with 10% FBS. After 24 h, cells were transfected with control or CD133 specific siRNA. 3 µl siRNA (10 µM) was diluted in 250 µl Opti-MEM reduced serum medium (Opti-MEM, Invitrogen) and mixed with 3 µl of Lipofectamine RNAiMAX pre-diluted in 250 µl Opti-MEM I. After 20 minutes incubation at room temperature, the complexes were added into the cells.

## Results

### Morphology of EH-CA1a and EH-CA1b Bile Duct Cancer Cell Lines

**Figure 1:**
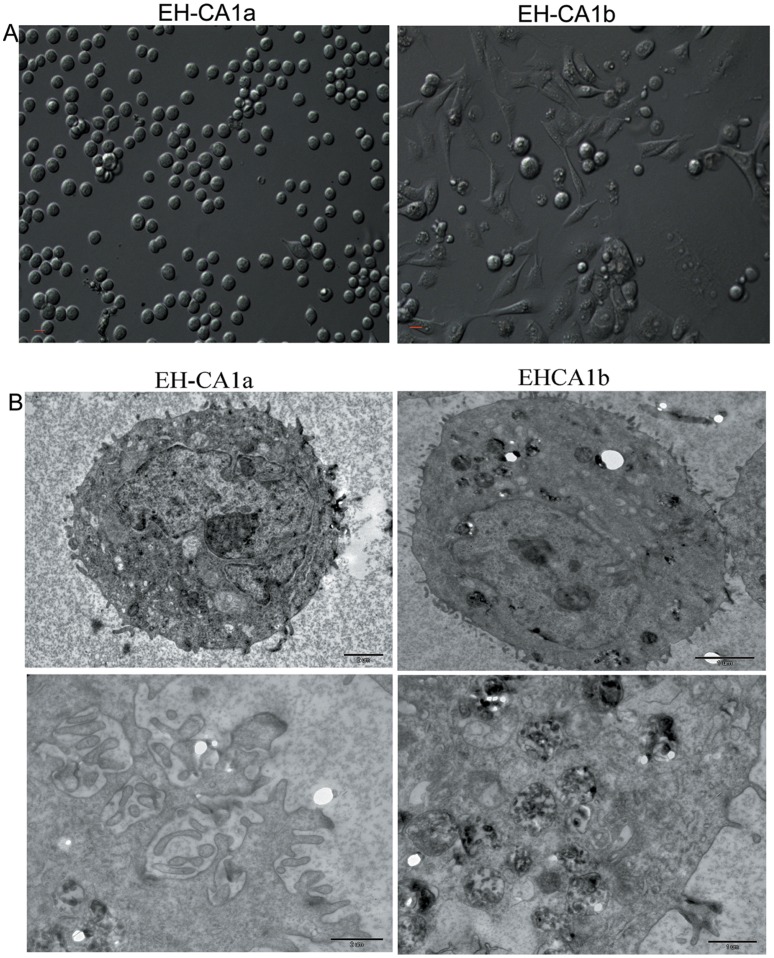
Morphology of EH-CA1a cells and EH-CA1b cells. (A). EH-CA1a cells are small, round, and grew in suspension, whereas EH-CA1b cells are relatively large, spindle-shaped, and grew as a monolayer (B) Transmission electron microscopy of EH-CA1a and EH-CA1b cell lines (TEM ×7500). Compared with EH-CA1b cells, EH-CA1a cells EH-CA1a cells have more microvillus on the cell surface.

Primary tumor cells obtained from BDC tissues were cultured in standard DMEM medium with 10% FBS (fetal bovine serum) for 5 days. Surprisingly, we found that two distinct cell subpopulations developed, and they were further subcloned based on their growth patterns. These two successive cell lines were named EH-CA1a and EH-CA1b. We succeeded in maintaining the EH-CA1a and EH-CA1b cells for more than 80 and 59 generations respectively in vitro with 10% FBS DMEM. The two established cell lines have different growth style and dissimilar morphology. We observed that EH-CA1a cells were round and suspended, whereas EH-CA1b cells were spindle-shaped and adherent (Figure 1A). During the following cultures, EH-CA1a and EH-CA1b had no evident changes in shape or growth pattern. A comparison of the ultrastructure between the two cell lines revealed that EH-CA1a cells had more intensive microvillus on the cell surfaces. This probably resulted from the deregulation of the actin and tubulin-based cytoskeleton, increased secretary vesicles in the cytoplasm (Figure 1B). The concentrations of the marker used for bile duct cancer diagnosis, CA19-9, in supernatant of EH-CA1a and EH-CA1b were 0.6 µg/ml and 8.6 µg/ml respectively (normal range, 0–35 µg/ml). The other markers such as α-fetoprotein (AFP) and carcinoembryonic antigen (CEA) were at the normal ranges in both cell lines.

### LOH/MSI of Selected Loci

**Figure 2:**
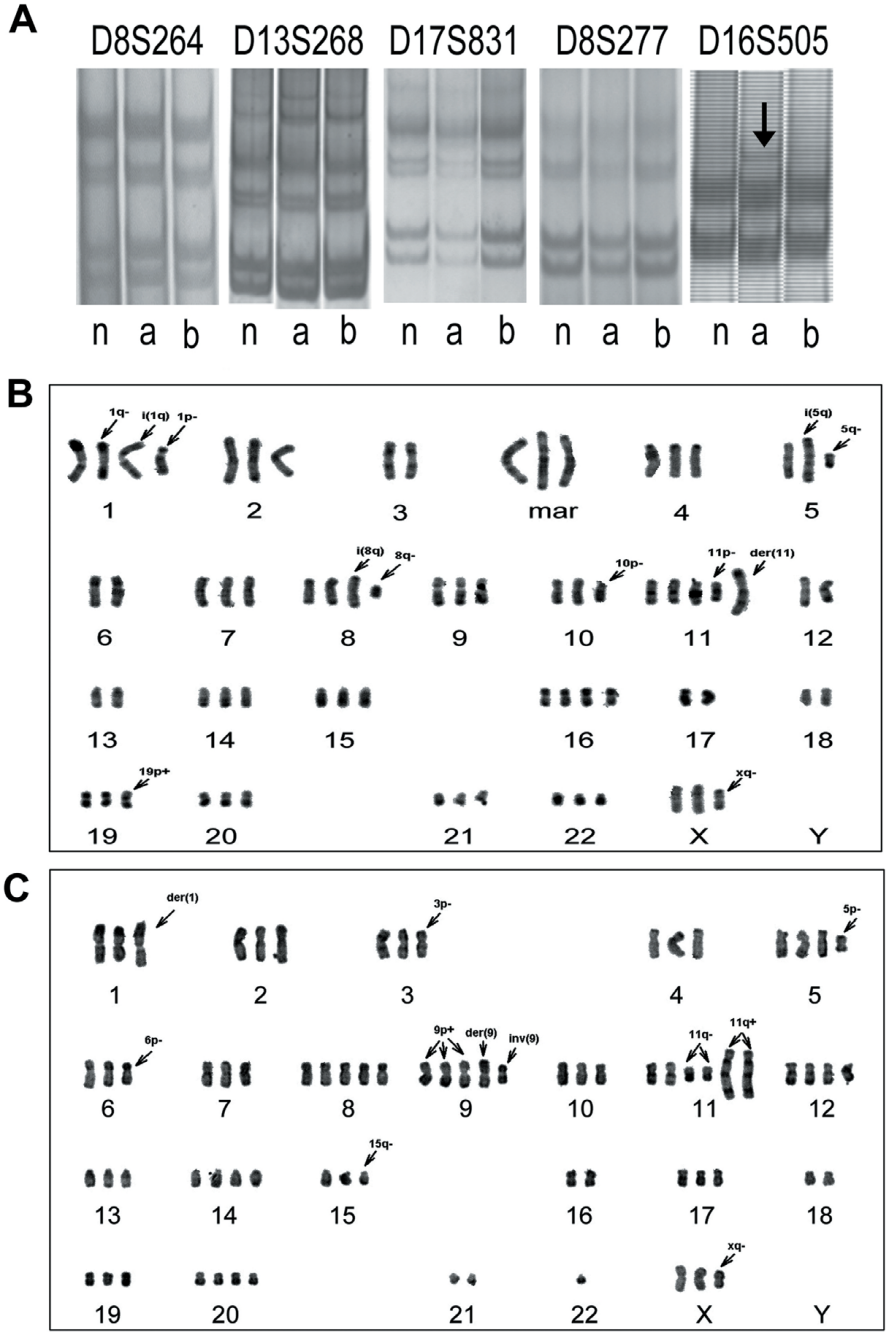
Characteristics of MSI of EH-CA1a cells and EH-CA1b cells. (A) Characteristics of MSI of the tumor and the normal bile duct epithelium. Compared with normal tissue (n) and EH-CA1b (b), EH-CA1a (a) had allelic expansion at the D16S505, where a band shift was detected (arrow). The alleles at D8S264, D8S277, D13S268 and D17S831 probes were present in both EH-CA1a and EH-CA1b cells, indicating that EH-CA1a and EH-CA1b cells lines are derived from the same patient. (B) G-banded karyotype of EH-CA1a, the photo showed an EH-CA1a cell containing 71 chromosomes. (C) G-banded karyotype of EH-CA1b, the photo showed an EH-CA1b cell with 73 chromosomes. The 17p was lost in EH-CA1a cells in comparison with EH-CA1b cells, indicating genetic heterogeneity of these two cells.

In order to confirm the same origin of the two cell lines, microsatellite instability (MSI) and loss of heterozygosity (LOH) at D8S264, D8S277, D13S268, D16S505, and D17S831, which locate on 8p23, 8p23, 13q14, 16q24 and 17p13 respectively, were performed on both normal bile duct epithelium of the patient and EH-CA1a, EH-CA1b cells by LOH/MSI assay [20]. Compared with normal bile duct epithelium and EH-CA1b, EH-CA1a cells showed an allelic expansion at the D16S505, this was displayed by one-band shift (arrow) (Figure 2A). The same sized alleles at D8S264, D8S268, D17S831 and D8S277 were found in EH-CA1a and EH-CA1b cells, indicating that both cell lines were derived from the same patient.

### Chromosome Analysis of EH-CA1a and EH-CA1b Cells

The results show that karyotype abnormalities in number and structure are present in both EH-CA1a and EH-CA1b cell lines. The chromosomal number varied from 68 to 73 (with a mode of 70) for EH-CA1a, and ranged from 68 to 80 (with a mode of 76) for EH-CA1b. Out of 50 chromosomes, 10 metaphase spreads were karyotyped along with major karyotypic features for EH-CA1a and EH-CA1b (Figure 2B, 2C). The arrowheads indicate rearranged chromosomes. The results indicate that there were at least three significant abnormalities: del(1)(pter→q21::?), i(1)(qter→q10::q10→qter), der(11)t(?::pter→qter::q13→qter) and der(?) were found invariably in EH-CA1a cells (30/30). Additional abnormalities include del(3)(qter→p23:), der(9)t(9qter→q10::14qter→q10), add(9)(qter→pter::?), del(11)(pter→q14:) and add(11)(pter→*qter*::?), which were found in all of the EH-CA1b cells. Other alterations and abnormalities were also frequently found in Table 2 and Table 3. The data indicated that these cells were heterogeneous at the chromosome level.

**Table 2:** Abnormal chromosomes for EH-CA1a and EH-CA1b cell lines.

EH-CA1a		EH-CA1b	
M1[1q−]	del(1)(pter→q21::?)	M1[der(1)]	der(1)t(1qter→p34::1q25→qter)
M2[i(1q)]	i(1)(qter→q10::q10→qter)	M2[3p−]	del(3)(qter→p23:)
M3[1p−]	del(1)(qter→p21:)	M3[5q−]	del(5)(pter→q14:)
M4[i(5q)]	i(5)(qter→q10::q10→qter)	M4[6p−]	del(6)(qter→p22:)
M5[5q−]	del(5)(pter→q13:)	M5[7q−]	del(7)(per→q34:)
M6[i(8q)]	i(8)t(qter→q10::q10→qter)	M6[8q−]	del(8)(pter→q21:)
M7[8q−]	del(8)(pter→q13:)	M7[der(9)]	der(9)t(9qter→q10::14qter→q10)
M8[10p+]	add(10)(qter→pter::?)	M8[9p+]	add(9)(qter→pter::?)
M9[10p−]	del(10)(qter→p12:)	M9[inv(9)]	inv(9)(pter→p13:q22→p13::q22→qter)
M11[der(11)]	der(11)t(?::pter→qter::q13→qter)	M10[10p−]	del(10)(qter→p12:)
M12[12p+]	add(12)(qter→pter::?)	M11[11q−]	del(11)(pter→q14:)
M13[13q−]	del(13)(pter→q14:)	M12[11q+]	add(11)(pter→qter::?)
M14[17p−]	del(17)(qter→p12:)	M13[15q−]	del(15)(pter→q15::q23→ater)
M15[19p+]	add(19)(qter→pter::?)	M14[xq−]	del(X)(pter→q24:)
M16[21q+]	add(21)(pter→qter::?)		
M17[xq−]	del(X)(pter→q24:)		
M18(mar)	der(?)		

**Table 3:** Frequency of abnormal chromosomes in 30 karyotype analyzed.

EH-CA1a					EH-CA1b			
Extra Chromsomes		Abnormal Chromsomes			Extra Chromsomes		Abnormal Chromsomes	
Chromsome	Frequency	Marker		Frequency	Chromsome	Frequency	Marker	Frequency
+2	29	M17	22		+1	27	M1	29
+4	30	M1	30		+2	19	M2 or M2*2	30
+5	27	M2	30		+3 or+3*2	30	M4	10
+7	30	+M3	21		+4 or+4*2	30	M6	15
+8	30	M4	29		+5*2 or+5*3	28	M7 or M7*2	30
+9	30	M5	11		+6	16	+M8 or M8*2	30
+10	11	M6	26		+7or+7*2	27	+M9	29
+11*2 or +11*3	30	M10	13		+8or+8*2 or+8*3	30	M10	14
+12	24	M11	30		+10 or+10*2	29	M11 or M11*2	30
+14	29	M12	25		+11 or+11*2	30	+M12 or+M12*2	30
+15 or +15*2	28	M15	18		+12 or+12*2	30	M13	21
+16 or+16*2	26	+M18*2	30		+13 or+13*2	28	M14	26
+17	22	M17	22		−14	10		
+19	30	M1	30		+15	22		
+20 or+20*2	29	M2	30		+16	18		
+22	28				+17	14		
+X	28				+19 or+19*2	26		
					+20 or+20*2	25		
					−22 or −22*2	29		
					+X	30		

### *In vitro* Proliferation and Hypoxia Tolerance of EH-CA1a and EH-CA1b Cells

**Figure 3:**
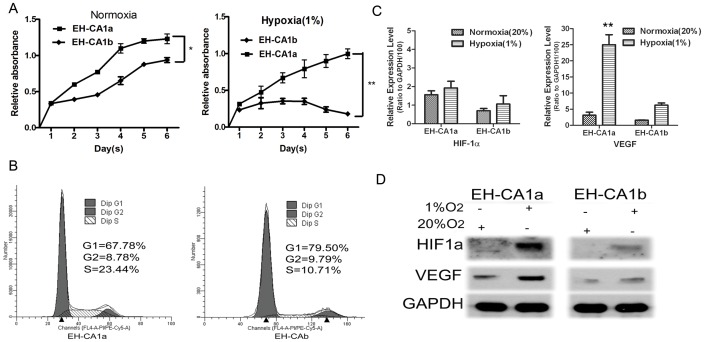
Proliferation and hypoxic resistance of EH-CA1a and EH-CA1b cells. (A) Cell proliferation of EH-CA1a and EH-CA1b. The curves showed that EH-CA1a grew slightly faster than EH-CA1b and was able to continue proliferating under 1% O2. (B) Cell cycle analysis of EH-CA1a and EH-CA1b. Ratio of G1/S results showed that EH-CA1a cells proliferated slightly faster than EH-CA1b cells. (C) Expression of HIF-1α and VEGF mRNA and (D) protein in EH-CA1a cells and EH-CA1b cells under normoxic and hypoxic conditions. Data was presented as ratio to mRNA level of GAPDH in each cell. (* P<0.05 and **p<0.01).

We first examined doubling growth time for the two cell lines. As shown in Figure 3A, EH-CA1a grew much faster than EH-CA1b in normoxic environment; their population doubling times are about 30 hours and 65 hours, respectively. We also observed their growth ability under hypoxic conditions. Surprisingly, we found that EH-CA1a can survive in the hypoxic conditions (1% O_2_), while nearly all of EH-CA1b cells were dead in the second day, demonstrating that EH-CA1b was unable to survive under hypoxic conditions (Figure 3A). Concurrently, cell cycle analysis shows that in normoxic conditions, about 30% of the EH-CA1a cells and 20% of the EH-CA1b cells were in the S-G2-M phases (Figure 3B). Hypoxia-inducible factor 1 (HIF-1) is a transcription factor that promotes angiogenesis, metabolic reprogramming, and other critical aspects of cancer biology (Hui et al 2002). Here we found that under normoxic conditions, the mRNA expression levels for HIF-1α and VEGF are nearly two-fold higher in EH-CA1a cells than that in EH-CA1b cells. Under the hypoxic conditions (1% oxygen), VEGF mRNA expression was dramatically increased by ∼7 times in EH-CA1a cells, while it was increased by ∼3 times in EH-CA1b cells in comparison with that under normoxic conditions (Figure 3C). Western blot analysis showed that, under hypoxic conditions, HIF1a and VEGF proteins significantly increased in EH-CA1a cells, and moderately increased in EH-CA1b cells. (Figure 3D).

### Colony Formation and Invasion Assays

**Figure 4:**
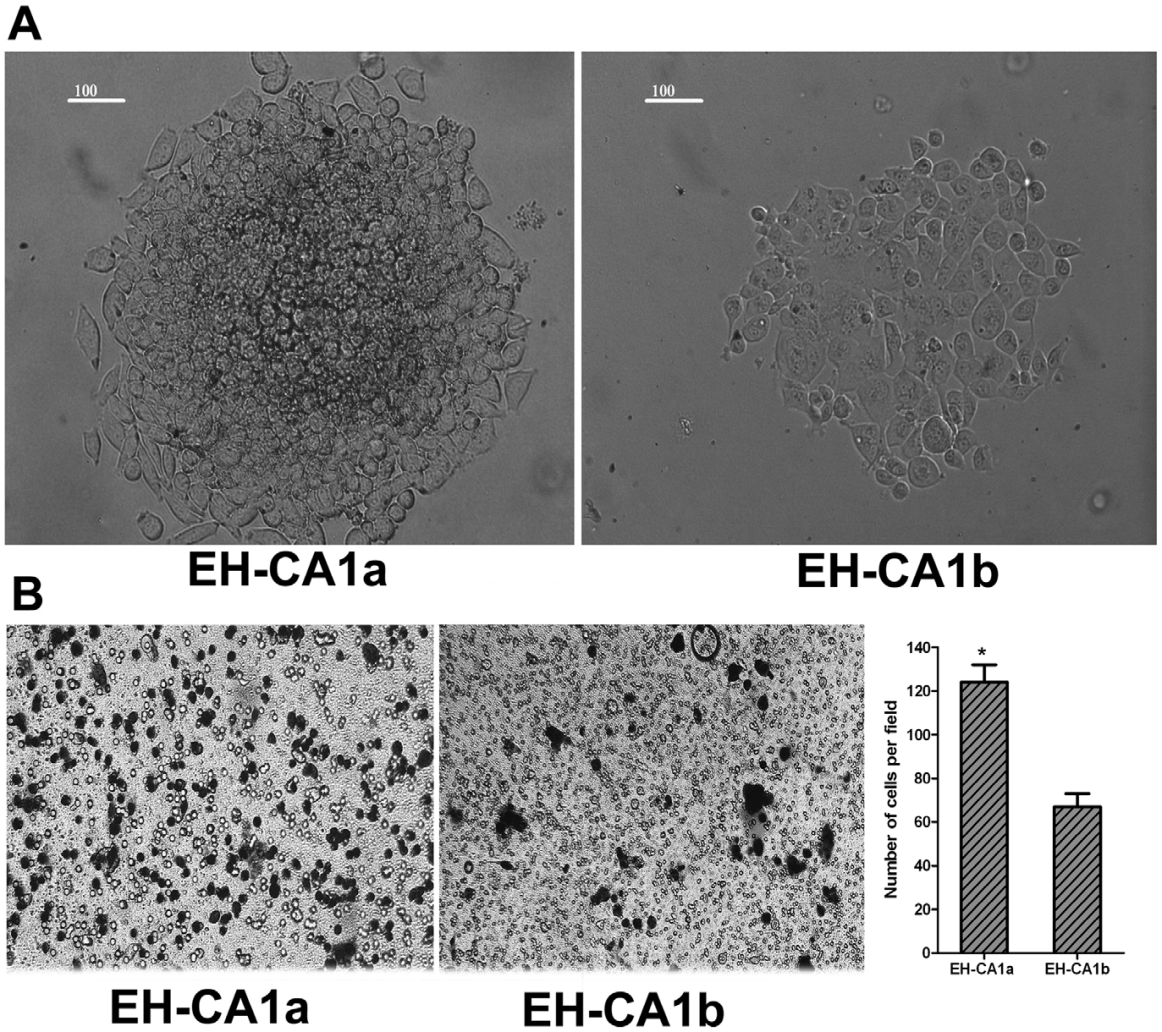
The colony formation and invasive potential of EH-CA1a cells and EH-CA1b cells. (A). Colony formation of EH-CA1a and EH-CA1b. Standard soft-agar assays were performed on these cells. Both cells efficiently formed cell colonies in soft agar; however, the number and size of colonies formed by EH-CA1a cells were much higher than EH-CA1b cells. (B) Invasive potential of EH-CA1a and EH-CA1b. The phase-contrast images of the invasion assay (left) showed that much more EH-CA1a cells were passed through the matrigel than EH-CA1b cells. Results represent means ± standard deviations from three independent experiments (*P<0.05).

Colonies were formed in both cell lines two weeks after plating 5,000 cells in soft agar. However, the number and size of colonies formed by EH-CA1a cells were much higher than EH-CA1b cells (Figure 4A). The phase-contrast images of the invasion assay (left) showed that much more EH-CA1a cells were passed through the matrigel than EH-CA1b cells (Figure 4B). Results represent means ± standard deviations from three independent experiments (* P<0.05).

### *In vivo* Tumorigenicity of EH-CA1a and EH-CA1b Cells

**Figure 5:**
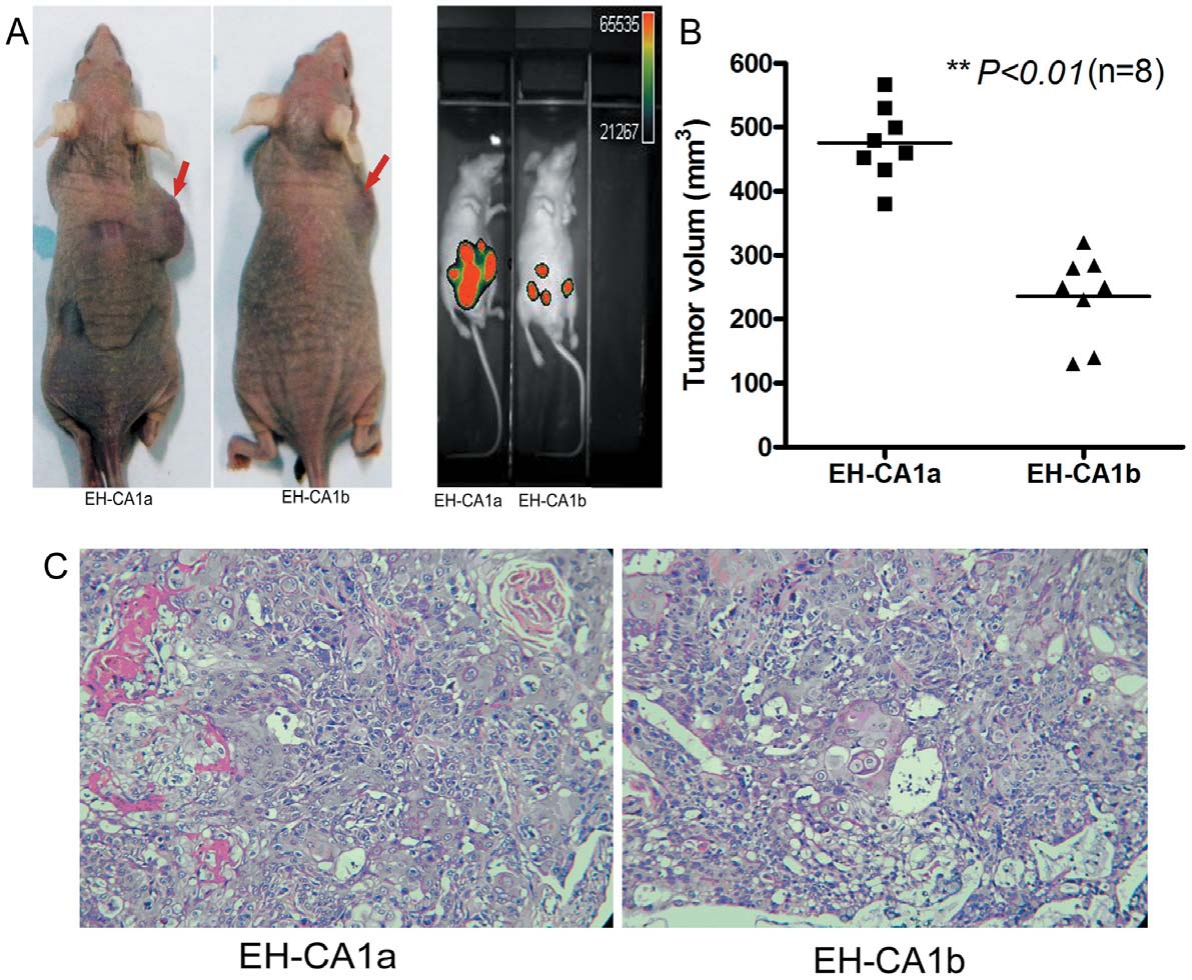
*In vivo* tumorigenicity of EH-CA1a cells and EH-CA1b cells. (A) Tumor xenograft of EH-CA1a cells and EH-CA1b cells. (Left) Xenograft tumor images showed that EH-CA1a cells had a much stronger *in vivo* subcutaneous tumorigenesis than EH-CA1b cells. Bioluminescent imaging images showed that the migration and expansion of EH-CA1a-Luc cells were much larger than that of EH-CA1b-Luc cells. (B) Volume of EH-CA1a cells and EH-CA1b tumor xenografts. Xenograft tumor volume of EH-CA1A was significantly higher than that of EH-CA1b (**p<0.01). (C) H & E staining of EH-CA1a and EH-CA1b tumor xenograft. H&E staining showed that both xenograft tumor tissues were adenocarcinoma; EH-CA1a xenograft tumor tissue was poorly differentiated; cell density of EH-CA1a xenograft tumor tissue was much higher, cancer cells had more irregular gland structures in comparison with EH-CA1b tissue.

In nude mice, both EH-CA1a and EH-CA1b cell lines have strong subcutaneous tumorgenicity; EH-CA1a cells exhibited a faster growth rate than EH-CA1b cells (Figure 5A). As we know, metastasis is the most important prognostic factor in cancer progression. Here we mimicked the in vivo process of cancer cell detachment and metastasis by inoculating EH-CA1a-Luc and EH-CA1b-Luc cells into mouse abdominal cavities; the colonization was monitored by bioluminescent imaging. Two weeks later, the bioluminescent intensity in mice inoculated with EH-CA1a cells was clearly greater than that in mice inoculated with EH-CA1b cells (Figure 5A). Furthermore, subcutaneous tumor volumes showed significant differences between the two groups *(P<0.01)* (Figure 5B), and the malignant tumor was confirmed by H&E examination (Figure 5C).

### *In vitro* Sensitivity of EH-CA1a and EH-CA1b Cells to Radiation and Chemotherapy

**Figure 6:**
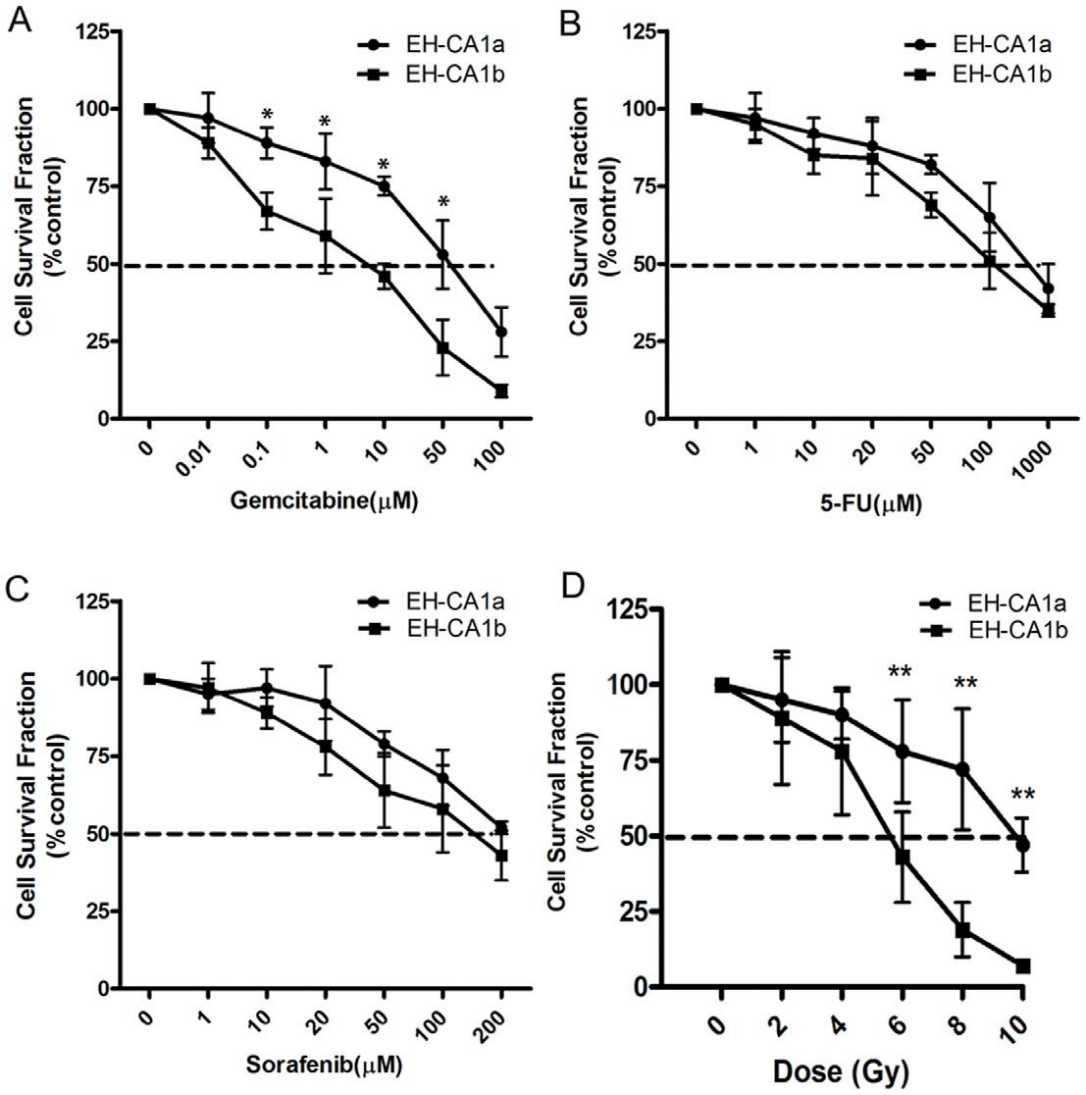
Sensitivity of EH-CA1a cells and EH-CA1b cells to anti-cancer drugs and radiation. Sensitivity of EH-CA1a cells and EH-CA1b cells to Gemcitabine, 5-Fu and sorafenib (A, B, C). The results showed that the resistance of EH-CA1 against gemcitabine was much higher than that of EH-CA1b cells. (D) Radiosensitivity of EH-CA1a cells and EH-CA1b cells. The results showed EH-CA1a was significantly less sensitive to radiotherapy than EH-CA1b cells. Data was presented as mean±SD of three independent experiments in quadruplicate. (*P<0.05 and **p<0.01).

Colony survival and MTT assays were used to determine the sensitivity of EH-CA1a and EH-CA1b cells to radiation and drugs including gemcitabine, 5-FU, and sorafenib. As shown in Figure 6, the viability of EH-CA1a and EH-CA1b cells can be inhibited by gemcitabine, 5-Fu, and sorafinib in a dose dependent manner. Interestingly, we also found that EH-CA1a cells exhibited significantly greater resistance to gemcitabine as opposed to EH-CA1b cells (Figure 6A). Both EH-CA1a and EH-CA1b cells showed less sensitivity to 5-Fu and sorafinib compared with Gemcitabine (Figure 6 B, C). However, we still found EH-CA1a cells to be more resistance to 5-Fu and sorafinib than EH-CA1b cells.

Radiotherapy is one of the most common methods we use in bile duct cancer treatment. Here we compared the sensitivity of EH-CA1a and EH-CA1b cells to radiotherapy. Results demonstrated that EH-CA1a cells have a higher radiation resistance than EH-CA1b and is consistent with the results of chemotherapy (Figure 6D).

### Differential Expression of EMT and Metastasis Relative Proteins

**Figure 7:**
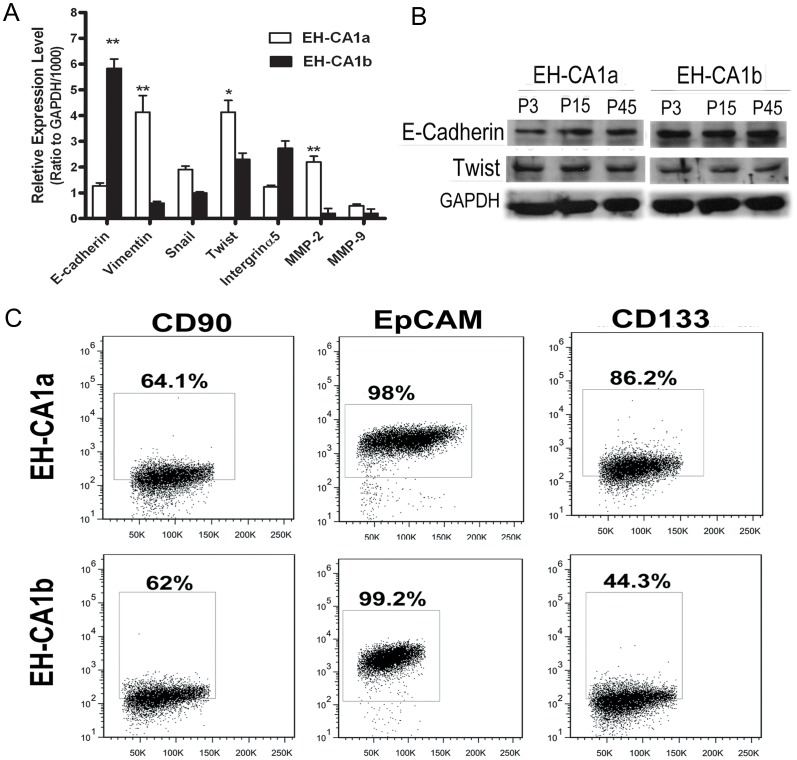
Differential expression of EMT, tumor metastasis related markers and cancer stem cell markers on EH-CA1a cells and EH-CA1b cells. (A). EMT and metastasis related genes expression in EH-CA1a and EH-CA1b.qRT-PCR analysis revealed that mRNA levels of Vimentin, Snail, Twist and MMP-9 were significantly higher in EH-CA1a cells than in EH-CA1b cells, while mRNA of E-cadherin in EH-CA1a cells was significantly lower than EH-CA1b cells. Data was presented as ratio to mRNA level of GAPDH in each cell. (*P<0.05 and **p<0.01). (B) Expression of E-cadherin and Twist in three different passages of EH-CA1a and EH-CA1b. (C) Expression of cancer stem cell markers in EH-CA1a and EH-CA1b cells. Flow cytometric analysis showed that 86.2% of EH-CA1a cells were CD133 positive, whereas 44.3% of EH-CA1b was CD133 positive. CD90 and EpCAM were similarly expressed in both cell lines.

Emerging evidence supports the relationship among EMT, tumor metastasis, drug resistance, and tumor recurrence [24]. In this study, different mRNA expression levels of EMT relative genes between two cell lines were detected. Results showed that E-cadherin mRNA expression was nearly four-fold lower in EH-CA1a cells compared with EH-CA1b cells, while Vimentin, Snail, Twist, MMP-2 and MMP-9 genes were higher expressed in EH-CA1a cells (Figure 7A). Consistently, the protein expression level of E-cadherin was lower, while the level of Twist was higher in EH-CA1a cells compared with that of EH-CA1b cells (Figure 7B). The difference in expression profiling of these EMT genes may partially determine the distinct malignancy of the two cell lines. We also found that the expression levels of E-cadherin and Twist were quite similar in three different passages of EH-CA1a and EH-CA1b.

### The Expression of Cancer Stem-cell Markers in EH-CA1a and EH-CA1b

Recently, more and more studies consider cancer stem cells to play key roles in cancer metastasis, recurrence, and drug resistance [25]. It is reported that only a few cancer cells possess the abilities of cancer stem cells and express the surface markers such as CD133, CD90 and EpCAM. The results (Figure 7C) showed that the percentages of cells positive for CD90 and EpCAM in both EH-CA1a and EH-CA1b cells were similar. However, the percentage of cells expressing CD133 in EH-CA1a was two times higher than that in EH-CA1b cells. To check the consistency of the expressions of these cellular surface proteins; we repeated the above experiment three times with differential passage of cells. Averages of the percentages of CD90-positive, EpCAM-positive and CD133-positive cells in three passages (8, 26 and 56) of EH-CA1a cells were 63.97±4.07%, 97.3±0.88% and 88.4±1.95%, respectively, while that in three passages (6, 22 and 52) of EH-CA1b cells were 65.33±3.8%, 97.8±1.78% and 45.67±1.27%, respectively. Statistics analysis showed the percentages of cells positive for above proteins in these three passages of both EH-CA1a and EH-CA1b cells were not significantly different. The above data suggested that the subpopulations in these two cell lines were quite stable during long-term *in vitro* culture.

### Knockdown of CD133 in EH-CA1a and EH-CA1b Cells Reduced the Invasive Potential and Increased the Sensitivities to Gemcitabine and Radiation

**Figure 8:**
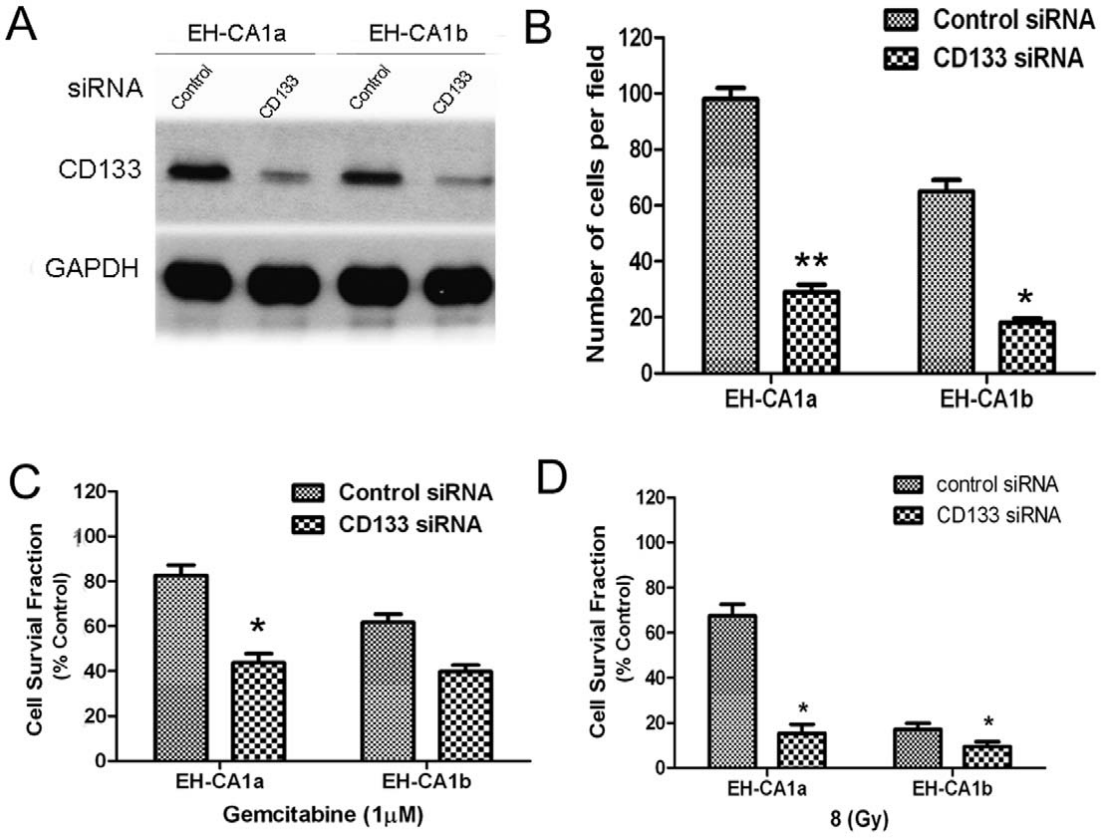
Knockdown of CD133 in EH-CA1a and EH-CA1b cells reduced invasive potential and increased sensitivities to gemcitabine and radiation. (A) Western blot of CD133. Control for scramble siRNA, CD133 for CD133 specific siRNA. At 72 h post-transfection of siRNA, cell pellets were collected for Western blot. (B) Knockdown of CD133 significantly decreased invasive cells in EH-CA1a and EH-CA1b cells; data is presented as the number of invasive cells in per field. Knockdown of CD133 increased sensitivity of both cells to gemcitabine (C) and radiation (D). Twenty-four hours after transfection with either control scrambled siRNA or CD133 specific siRNA, cells were subjected to matrigel invasion assay for 24 hours; or cells were exposed to gemcitabine for 72 hours; or cells were subjected to 8 Gy radiation, and then cultured for 72 hours. Cell death was measured by MTT assay. Mean values ± S.D. are shown. *P<0.05, **P<0.01, compared with control scrambled siRNA transfection.

Expression of CD133 was considered relates with stronger tumorigenesis invasion, and chemo-radiotherapy resistance in many kinds of cancers, such as liver cancer [26], glioblastoma [27], and pancreatic cancer [28]. In order to study contribution of CD133 to malignant potentials of EH-CA1a and EH-CA1b cells, the CD133 gene was silenced using a small interfering RNA. As shown in Figure 8A, the level of CD133 was higher in EH-CA1a than EH-CA1b cells, CD133 siRNA substantially decreased CD133 protein in both cells. We found that knockdown of CD133 rarely suppressed the *in vitro* proliferation of both cancer cells, while knockdown of CD133 significantly decreased the invasive potentials of both cancer cells *(p<0.05)* (Figure 8B). The sensitivity to 1 µM gemcitabine significantly increased in the CD133-attenuated EH-CA1a and EH-CA1b cells compared with control cells transfected with scramble siRNA *(p<0.05)*, especially in EH-CA1a cells (Figure 8C). Similarly, the sensitivity to 8 Gy radiation was significantly increased in EH-CA1a cells after knockdown of CD133 *(p<0.05)*, while moderately increased in EH-CA1b cells (Figure 8D). The above data indicated that CD133 may play important roles in the malignancy of both cancer cells.

## Discussion

The establishment of multiple heterogeneous cell lines for BDC will have significant implications not only for basic BDC research, but also for the clinical development of novel therapeutic approaches. However, only a few bile duct cancer cell lines are currently available, since the success rate for establishment of bile duct carcinoma cell lines is extremely low, commonly less than 1% [29]. To our best knowledge, BDC cell lines established from the same tumor foci reflecting intratumoral heterogeneity have not been documented. In the present study, we successfully established and characterized two cell lines, namely EH-CA1a and EH-CA1b, from primary tumor foci of a pathologically proven BDC tissue, which displayed distinct intratumoral heterogeneities in many aspects of tumor biology. We consider that the established EH-CA1a and EH-CA1b cell lines would shed the light on intratumoral heterogeneity and provide a potential tool to develop novel therapeutic approaches for BDC.

Increasing studies have revealed that intratumoral cells are highly heterogeneous genetically. For example, a most recent study showed that ccRRC cells in the same patient have different genomic landscape at the single cell level [30]. Another study found that spatially separate regions of the same clear cell renal carcinoma harbors heterogeneous somatic mutations and chromosomal imbalances, providing the molecular evidence for intratumoral heterogeneity [9], [30]. In the present study, we found that the two cell lines have distinct karyotype. For instance, karyotype analysis showed that both cells contained chromosome marker del (10)(qter→p12:) in regions where tumor suppressor gene PTEN is located. Loss of PTEN gene was demonstrated in carcinoma cells, indicating poor prognosis in bile duct cancer [31], prostate cancer [32], and lung cancer [33]. However, the loss of 17p was specific to EH-CA1a, indicating genetic heterogeneity of these two cells.

Prominent heterogeneities of EH-CA1a and EH-CA1b are their growth pattern and outer shapes morphology. EH-CA1a grew as floating aggregates, while EH-CA1b grew adherently as a monolayer in the same culture medium. Floating growth patterns and small cell size for cells from epithelial tumors may facilitate tumor cells in separating from its original foci, traversing through the blood vessels, evading the immune attacks in the circulation during tumor cell transport, and reducing the mechanical resistance during penetration in the target tissue. All these in vitro cellular studies indicate that EH-CA1a cells possess stronger tumorgenicity and more metastatic potential.

The adaptation of tumor cells to hypoxia is a crucial driving force in the clonal selection that results in a more aggressive and therapy-resistant tumor phenotype [34]. HIF-1α is highly expressed in human cancers as a result of intratumoral hypoxia as well as genetic alterations, such as gain-of-function mutations in oncogenes (for example, ERBB2) and loss-of-function mutations in tumour-suppressor genes (for example, VHL and PTEN) [35]. HIF-1α high expression is associated with treatment failure and increased mortality [36]. EH-CA1a, hypoxia-resistant cell line, showed aggressive and strong drug and radiation resistance. We also found that EH-CA1a cells have a higher basic HIF-1α and VEGF expression than EH-CA1b cells under normoxic condition. DL Roberts and colleagues [37] demonstrated that activation of HIF-1 provoked changes in apoptotic pathway regulatory protein levels that affect drug sensitivity.

EMT is a key step toward cancer metastasis, and many major transcription factors are involved [38]. Here we analyzed the expression level of EMT related inducers/regulators, Snail, Twist, matrix metalloproteinase (MMPs)-2, -9 [39], [40], [41], [42], E-cadherin, Integrinα5, and VEGF of EH-CA1a/b cell lines [43], [44]. E-cadherin is an adhesion protein that plays a central role in epithelial morphogenesis, and the expression of this protein is downregulated during the acquisition of metastatic potential at late stages of epithelial tumor progression [45]. In a present study, we found EMT markers such as Vimentin, Snail Twist, MMP-2 and MMP-9 to be significantly higher, while E-cadherin was lower in EH-CA1a than EH-CA1b, which indicated that EH-CA1a cell lines are more likely to invade or metastasize [46], [47].

Furthermore, we characterized differential expressions of cancer stem cell markers in these two cell lines. We surprisingly found that both EH-CA1a and EH-CA1b cell lines have a high population of cancer stem cell-like cells. This indicates that these two cell lines may possess obvious cancer stem cell features, which may explain their strong potential for *in vitro* clone formation and *in vivo* tumorigenesis. Tumor cells with high levels of cancer stem cell marker CD133 usually have stronger tumorigenesis [26], invasion, and chemo-radiotherapy resistance. Consistently, the CD133-positive cells of EH-CA1a were nearly double in number than EH-CA1b cells.

Knockdown of CD133 in EH-CA1a and EH-CA1b significantly decreased invasive potentials of both cells and increased sensitivities of both cells to chemoradiotherapy, which may indicate the important roles of CD133 in the malignant characteristics of these two cancer cells. We further found that after knockdown of CD133, the sensitivity of EH-CA1a to chemoradiotherapy became closer to that of EH-CA1b cells. Therefore, we considered that differential expression of CD133 protein may partially account for the intratumoral heterogeneities of the BDC.

Resistance to chemotherapy is the primary cause for treatment failure in the vast majority of patients with advanced and inoperable bile duct cancer [48]. Multiple mechanisms are responsible for chemo/radio-resistant in cancer [49]. We considered that significant difference of EH-CA1a and EH-CA1b in response to gemcitabine and radiotherapy may further indicate the intratumoral heterogeneity, and might be determined by other intratumoral genetic heterogeneities of the BDC.

In conclusion, we have established two new bile duct cancer cell lines with distinctive characteristics, namely EH-CA1a and EH-CA1b, from primary tumor foci of a pathologically proven BDC tissue, which reflect highly intratumoral heterogeneity of BDC. They may potentially facilitate to the development of novel therapeutic approaches for BDC.
